# Synergistic Effect of Sodium Acetate and Sodium Butyrate in Ameliorating Ethanol‐Induced Hepatic Inflammation Through Modulation of the NF‐κB Signaling Pathway

**DOI:** 10.1155/mi/5835567

**Published:** 2026-06-20

**Authors:** Manali Patel, Hiral Aghara, Prashsti Chadha, Harshrajsinh Solanki, Dhrubjyoti Sharma, Vijay Thiruvenkatam, Palash Mandal

**Affiliations:** ^1^ Department of Biological Sciences, P. D. Patel Institute of Applied Sciences, Charotar University of Science and Technology, Changa, Anand, Gujarat, India, charusat.ac.in; ^2^ Department of Biological Sciences and Engineering, Indian Institute of Technology Gandhinagar, Palaj, Gandhinagar, Gujarat, India, iitgn.ac.in

## Abstract

Chronic ethanol exposure activates inflammatory signaling pathways and inflicts hepatocellular damage, leading to alcohol‐associated liver diseases (ALDs). ALD is one of the major causes of global burden, yet there are no FDA‐approved treatment options available. This study evaluates the hepatoprotective effects of short‐chain fatty acids (SCFAs), mainly sodium acetate (NaA) and sodium butyrate (NaB), against ethanol‐induced inflammation and oxidative stress in both in vitro (Buffalo Rat Liver‐3A [BRL3A]) and in vivo (male Wistar rats) models. The treatment of NaA and NaB and their combination was given to the cell lines where maximum viability was observed at concentrations of 1.5 mM, 5 mM, and 0.1 mM + 1 mM, respectively. Additionally, reactive oxygen species (ROS) and nuclear morphology were assessed by fluorescent staining. For in vivo samples, the hepatic injury was analyzed by serum biochemical markers. Furthermore, hematoxylin and eosin (H&E) staining and immunohistochemistry (IHC) staining were employed, which provided structural and immunological alterations in hepatic tissue. RT‐qPCR profiled the expression levels of various pro‐inflammatory and anti‐inflammatory cytokines, as well as cytochrome P450 E1 (CYP2E1) and antioxidative stress markers. Moreover, enzyme‐linked immunosorbent assay (ELISA) quantified the essential protein targets such as TNF‐α, MCP‐1, IL‐1β, IL‐6, HO‐1, and Nrf2. The administration of NaA, NaB, and their combination resulted in reduced ROS levels and expression of pro‐inflammatory cytokines, preserved nuclear integrity, and neutrophil infiltration. These findings were further confirmed by in silico analysis and conserved amino acid interactions, and the affinities of NaA and NaB for TNF‐α and MCP‐1 were observed as compared to established inhibitors or activators. This study is the first demonstration to report the synergistic effects of NaA and NaB on the feedback loop of the nuclear factor kappa B (NF‐κB) signaling pathway, suggesting their potential as promising therapeutic candidates for alleviating alcohol‐induced hepatic damage.

## 1. Introduction

Alcohol‐associated liver disease (ALD) represents a critical global health issue, accounting for 5.1% of liver‐related morbidity and mortality [1, 2]. The alcohol‐induced liver injury involves a spectrum of disease, where the trajectory expands from alcoholic steatosis and hepatitis to cirrhosis and end‐stage liver failure. The early stages of liver injury result in fat accumulation and low‐grade inflammation; however, persistent alcohol consumption leads to severe hepatic damage and activation of hepatic stellate cells (HSC) [3]. Multiple experimental and clinical studies identified oxidative stress and inflammation as two fundamental contributors to the pathological alteration of ALD [4]. The ALD pathogenesis is rather complex, including direct modification of the inflammatory signaling pathway in the liver and triggering significant release of pro‐inflammatory cytokines. Among several signaling pathways, the nuclear factor kappa B (NF‐κB) signaling cascade plays a pivotal role in induce inflammation [5]. The NF‐κB pathway is activated by several inflammatory cytokines, which in turn regulate the expression of numerous genes associated with innate and adaptive immunity [6, 7].

NF‐κB subunits are prominently activated at inflammatory sites across various diseases. Under homeostatic conditions, NF‐κB is sequestered by IκB protein in the cytoplasm [8]. However, during substantial cellular distress, such as ALD, hepatocytes display elevated NF‐κB expression and promote immunocytes recruitment to stimulate the secretion of key pro‐inflammatory cytokines such as interleukin‐1, interleukin‐6, interleukin‐8, and tumor necrosis factor‐α [9]. Concurrently, the released pro‐inflammatory cytokines further influence NF‐κB activation, establishing a feedback loop that accelerates NF‐κB signaling by degrading IκBα complex [10]. Moreover, TNF‐α, IL‐6, and IL‐1β also engage in cross‐talk with tumor necrosis factor receptors to enhance the onset of NF‐κB expression [11]. Additionally, chemokines, such as monocyte chemoattractant protein‐1, also perturb a critical role to amplify this signaling pathway by attracting immune cells [12]. Notably, cytochrome P450 E1 (CYP2E1), an enzyme involved in metabolizing the excessive alcohol intake, can indirectly contribute in activating NF‐κB by generating reactive oxygen species (ROS), which serves as a secondary messenger to stimulate this pathway [13].

As the occurrence of ALD among the population continues to rise, a diverse range of therapeutics are developing beyond conventional interventions. One of these interventions includes gut microbiota and their metabolites that are increasingly acknowledged for their immunomodulatory role in the pathogenesis of ALD [14, 15]. The fermentation of dietary fibers and resistant starch by gut microbes produced short‐chain fatty acids (SCFAs), primarily acetate (C2), propionate (C3), and butyrate (C4) [16]. These SCFAs are predominantly responsible for providing energy to colonocytes, maintaining gut homeostasis, and promoting mucus production [17, 18]. In the proximal colon, SCFAs exist in anionic form and are transported across the gut lumen by monocarboxylate transporter 1 (MCT1) and sodium‐coupled MCT1 [19]. Once absorbed, SCFAs enter the portal circulation and are rapidly metabolized by hepatocytes [20]. Among all, the dietary sodium acetate (NaA) and sodium butyrate (NaB) have emerged as essential metabolites, exerting immunomodulatory properties to influence and mitigate ALD‐associated inflammatory pathways [21].

Multiple pieces of evidence have uncovered the role of NaA and NaB in counteracting inflammation by immune regulation [22–24]. These SCFAs influence local and systemic immune responses by governing the synthesis and release of cytokines and chemokines. As indicated in one of the studies, NaB inhibited pro‐inflammatory cytokines and chemokines in colitis patients by regulating neutrophils through the HDAC‐dependent pathway [21]. Similarly, NaA decreased neutrophil infiltration and alleviated pancreatitis in mice. Another research found that at 30 mM, both SCFAs inhibited TNF‐α release in LPS‐stimulated human and rat neutrophils, an effect that appears to involve the attenuation of NF‐κB activation [25]. Furthermore, evidence suggests the role of these SCFAs in the liver during ALD by diminishing oxidative stress through Nrf2‐regulated antioxidant pathways [26].

Although considerable evidence indicates the anti‐inflammatory and antioxidant properties of NaA and NaB, their possible synergistic efficacy in preventing ethanol‐induced hepatic damage remains insufficiently characterized. Presently, no approved medication exists for alcohol‐induced liver damage. Therefore, this study investigates the hepatoprotective potential of NaA, NaB, and their combination against alcohol‐induced liver injury, employing both in vitro (Buffalo Rat Liver‐3A [BRL3A] cells) and in vivo (male Wistar rat) models.

## 2. Materials and Methods

### 2.1. Materials

NaA and NaB were purchased from HiMedia (Mumbai, India). Minimal essential medium (MEM), antibiotic solution (mention composition), 1x phosphate‐buffered saline (PBS), fetal bovine serum (FBS), 3‐[4,5‐dimethylthiazol‐2‐yl]−2,5‐diphenyl tetrazolium bromide (MTT), and rat feed components were also obtained from HiMedia (Mumbai, India). 4′, 6‐diamidino‐2‐phenylindole (DAPI) and 2′, 7′‐dichlorodihydrofluorescein diacetate (H2‐DCFDA) were purchased from Merck (NJ, USA). Acridine orange (AO) and ethidium bromide (EtBr) were sourced from SRL (Mumbai, India). The gene primers for RT‐qPCR were acquired from Merck (NJ, USA) and Barcode Biosciences (Bangalore, India). For RNA isolation, cDNA synthesis, and qPCR, RNAisoplus (Takara Bio Sciences, Japan), Verso cDNA synthesis kit AB1453A) (Thermo Fisher, MA, USA), and PowerUp SYBR Green master mix (A25742) (Thermo Fisher, MA, USA) were used, respectively. Serum components for blood cholesterol, alanine aminotransferase (ALT), aspartate aminotransferase (AST), and triglycerides were obtained from Pariksha BioTech (Hyderabad, India). The gamma‐glutamyl transferase (GGT) kit was purchased from Coral Clinical System (Goa, India). Antibodies for immunohistochemical staining were obtained from Thermo Fisher Scientific (CYP2E1 Antibody PA5−35251) and Agilent Technologies (Envision HRP, Dako). Enzyme‐linked immunosorbent assay (ELISA) kits for rat TNF‐α (Cat. No. ELR‐TNF‐α−1), rat MCP‐1 (Cat. No. ELR‐MCP‐1), rat IL6 (Cat. No. ELR‐IL‐6), rat IL‐1β (Cat. No. ELR‐IL1‐β) and rat HO‐1 (Cat. No. ORB411280) and Nrf2 (Cat. No. ORB781880) were purchased from Biorbyt LLC (North Carolina, USA).

Table 1 elucidates the experimental groups of in vitro and in vivo models, providing information regarding the treatment groups, their respective dosages, durations, and objectives.

**Table 1:** Experimental cohorts of in vitro and in vivo studies with their relevant information.

Number	Group number	Group name	Treatment description	Dose/concentration	Duration	Purpose
1.	G1a	Control	No treatment given in for in vitro assays	NA	NA	Baseline comparison
2.	G1b	Pair fed	An isocaloric diet given to animals	NA	NA	Baseline comparison
3.	G2	Disease model	Induced with ethanol	In vitro dose: 250 mM In vivo dose: 5% everyday with 31.5% binge diet twice (11th day and 22nd day)	In vitro: 24 h In vivo: 26 days	Disease induction
4.	G3	NaA treatment	Disease + sodium acetate	In vitro dose: 250 mM ethanol + 1.5 mM NaA In vivo dose: 5% everyday ethanol + 150 mg/kg NaA	In vitro: 24 h In vivo: 26 days	Evaluate NaA effect
5.	G4	NaB treatment	Disease + sodium butyrate	In vitro dose: 250 mM ethanol + 5 mM NaB In vivo dose: 5% everyday ethanol + 300 mg/kg NaA	In vitro: 24 h In vivo: 26 days	Evaluate NaB effect
6.	G5	Combination NaA + NaB treatment	Disease + combined NaA and NaB	In vitro dose: 250 mM ethanol + (0.1 + 1 mM) In vivo dose: 5% everyday ethanol + (50 + 150) mg/kg NaA and NaB, respectively	In vitro: 24 h In vivo: 26 days	Synergistic effect

### 2.2. In Vitro Study

A healthy BRL3A cell line, derived from male BRL, exhibiting a fibroblast phenotype, was obtained from the National Centre for Cell Science (NCCS) in Pune, India. The RRID is CVCL_0606, which was obtained in January 2024. Since rat cell lines do not have a standardized international authentication database, species‐specific PCR was performed as described in [27], and no cross‐species contamination was identified. BLR3A cells are not listed as a misidentified or contaminated cell line in the ICLAC. The BRL3A cells were cultured in a MEM medium containing 10% FBS and 1% antibiotic solution. The cell line was maintained at a temperature of 37°C with a continuous supply of 5% CO_2_ in a water‐jacketed incubator (Thermo Fisher Scientific, MA, USA). Cells were regularly passaged, and the culture medium was replaced as necessary. All experiments were performed using cells between passage numbers 20 and 27. The cell line was screened for mycoplasma contamination using PCR‐based detection, and the test results were negative before performing the described experiments. The seeding densities for each assay were as follows: 96‐well plate, 2 × 10^4^ cells/well; 6‐well plate, 7 × 10^5^ cells/well. All experimental procedures were conducted in a 2% serum medium. NaA and NaB were prepared in Milli‐Q water. All assays were performed in triplicate.

#### 2.2.1. Cell Viability Assay

To determine the maximum dosages of NaA and NaB that maintain the highest cellular viability, an MTT assay was conducted. Cells were seeded into a 96‐well plate and allowed to adhere for 20 h. The following day, the treatment of NaA (0.5–5 mM), NaB (0.5–20 mM), and a combination of both compounds was carried out at low (0.1 + 1 mM), medium (1 + 2.5 mM), and high (2.5 + 7.5 mM) doses (Table 1). A dose of 250 mM of ethanol was used for cotreatment (Table 1). Post 24 h of treatment, MTT was added to achieve a final concentration of 0.5 mg/mL, and the plates were incubated in the dark at 37°C for 3 h. Later, the media was aspirated, and the formazan crystals were dissolved in 100 µL of DMSO. Absorbance was measured at 570 nm using a multimode reader (Perkin Elmer, MA, USA), and cell viability was calculated according to the following formula:

Cell viability %=As/Ac×100,

whereas As = absorbance of test and Ac = absorbance of control.

#### 2.2.2. DAPI Staining to Evaluate Nuclear Integrity

To evaluate the impact of ethanol, NaA, and NaB on nuclear integrity, DAPI staining was conducted. For this staining, cells were seeded within a 96‐well plate, following with the treatments of 250 mM ethanol, 1.5 mM NaA, 5 mM NaB, and a combination of NaA and NaB at low doses (0.1 + 1 mM). Following a 24‐h incubation period, the cells were fixed using 4% neutral buffered formalin, washed, and then incubated with DAPI (final concentration 20 µM) in darkness for 20 min. Finally, the cells were washed with 1x PBS and examined under a fluorescence inverted microscope (Nikon Eclipse 3000, Tokyo, Japan) equipped with a DAPI filter (405 nm).

#### 2.2.3. ROS Estimation

ROS generation is a key feature of alcohol metabolism. To assess ROS‐mediated cell death, we performed the H2‐DCFDA assay. Cells were seeded and treated as described in the previous section. Afterwards, they were fixed with 4% neutral buffered formalin. The cells were then stained with 20 µM H2‐DCFDA and incubated in the dark for 45 min. Finally, the cells were washed, and their fluorescence was measured using a multimode reader (Perkin Elmer, MA, USA) at 485/535 nm.

#### 2.2.4. AO/EtBr Dual Staining to Assess Cell Death

AO/EtBr dual staining was employed to evaluate cell death, nuclear morphological alterations, and apoptotic body formation. Cells were seeded and treated according to the methods outlined earlier. After 24 h, they were washed with 1x PBS and stained with a 100 µg/mL AO/EtBr solution. Immediately afterward, they were examined using a fluorescence inverted microscope (Nikon Eclipse 3000, Tokyo, Japan) with FITC (495/520 nm) and TRITC filters (550/573 nm).

### 2.3. In Vivo Studies

#### 2.3.1. Animal Experimental Design

Animal experiments were conducted in accordance with the standard ethical ARRIVE (Animal Research: Reporting of In Vivo Experiments) guidelines and received approval (RPCP/IAEC/2024‐25/R16) from the Institutional Ethics Committee of the Charotar University of Science and Technology. Almost 8‐ to 10‐week‐old male Wistar rats (weighing between 165 and 190 g) were obtained from Zydus Research Centre, Moraiya, Ahmedabad. All animals were incorporated, their dissection was conducted in accordance with the prescribed guidelines, and no animals were excluded from the study. The rats were housed under ambient conditions (25 ± 2°C with a relative humidity of 55% ± 5%) with two rats per cage. They were fed a standard chow diet with ad libitum access to water and acclimatized for 1 week. The rats were maintained on a 12 h light/dark cycle throughout the duration of the study. Following acclimatization, the animals were allocated to various experimental groups by a randomized computer‐based method. Histological and biochemical analyses were conducted blinded to group assignment to avoid bias. The animals were randomly assigned to six groups (*n* = 6): Control (NC), Pair‐fed (PF), Ethanol‐fed (disease control, E), NaA treatment (150 mg/kg), NaB treatment (300 mg/kg), and Combination treatment (50 mg/kg + 150 mg/kg) (Table 1) where the respective treatments were given by oral gavage. The sample size (*n* = 6 per group) was governed based on previously published studies that utilized comparable experimental models, in which this group size was adequate to identify significant biochemical and histological variations. The sample size was also chosen to minimize animal use in accordance with ethical guidelines. A formal power analysis was not performed. The PF group received an isocaloric maltodextrin Lieber‐DeCarli diet. The concentrations of ethanol and maltodextrin were gradually increased over the initial 5 days, starting at 1% and reaching 5% by the fifth day, thereafter remaining at 5% for the remainder of the study. Additionally, a binge diet consisting of 31.5% ethanol for two consecutive days was administered on the 11th and 22nd days to simulate normal drinking conditions [28].

#### 2.3.2. Histomorphology

##### 2.3.2.1. Liver Weight and Appearance

Post dissection, the liver samples were collected and weighed. Their size and appearance were noted and compared among all groups.

##### 2.3.2.2. Histopathological Analysis

The tissue samples were stored in 10% neutral buffered formalin. Sample preparation follows several steps. Initially, the samples were embedded in paraffin wax, which was washed for 2 h under running water. The blocks were then dehydrated through increasing the concentration of isopropyl alcohol in a series of 30%, 70%, 90%, and 100%. Later, the blocks were deparaffinized with xylene, followed by impregnation in molten paraffin wax. Thin sections of the tissues with a 4–5 µm size were sectioned using an automated rotary microtome (Leica RM2255). These sections were mounted on slides coated with poly‐L‐lysine (0.1% w/v in H_2_O) and stained with hematoxylin and eosin (H&E) as counterstain by a Gemini AS Automated Slide Stainer (Thermo Scientific). The stained slides were further examined under a phase contrast microscope at 40x magnification.

##### 2.3.2.3. Immunohistochemical Observation

The tissue preparation and fixation were analogous to the procedure of H&E staining. The sections were then deparaffinized and rehydrated. For antigen retrieval, the autoclave technique was used. After the complete autoclave cycle, the sections were incubated for 2 min in an antigen retrieval buffer at pH 9.0. Following the incubation, the sections were washed with tris‐saline buffer (TBS) at pH 7.4 and incubated in 3% hydrogen peroxide for 30 min to inhibit the endogenous peroxidase activity. The sections were further incubated with primary antibodies (Thermo Scientific, MA5−11883, 1:200) and rinsed with TBS. Subsequently, a 30‐min incubation was given in perxodiase/DAB, rabbit/mouse (Agilent, Dako, Denmark) in the EnVision Detection System. Finally, counterstain with Mayer’s hematoxylin was used to stain the sections.

##### 2.3.2.4. Measurement of Serum Liver Damage Indices

The serum biochemical markers, such as GGT, AST, and ALT, are employed to evaluate hepatocellular injury. Additionally, lipid metabolism impairment is assessed by serum TG and cholesterol levels. All assays were performed according to the manufacturer’s instructions provided in the commercially available kits. All procedures were performed in triplicate.

##### 2.3.2.5. Protein Isolation and ELISA

For protein analysis, 200 mg of tissue was thawed and homogenized in 500 μL of RIPA buffer [29] on ice. Subsequently, the samples were stored at −20°C for 2 h and then thawed and centrifuged at 12,000 rpm for 20 min at 4°C. The resulting supernatants, containing the proteins, were collected and subjected to ELISA for the detection of TNF‐α, MCP‐1, IL‐1β, IL‐6, HO‐1, and Nrf2. The specific ELISA kit employed was used in accordance with the manufacturer’s instructions.

### 2.4. Gene Expression Study

For in vitro gene expression analysis, cells were seeded in 6‐well plates. After 24 h, the cells were treated with ethanol, NaA, NaB, and their combination. For in vivo assays, a 200 mg liver tissue sample was homogenized. Total RNA from cells and tissues was extracted using RNA Isoplus (Takara) and reverse‐transcribed into cDNA with the Verso cDNA Synthesis Kit (Thermo Fisher). The targeted genes for both in vitro and in vivo analyses included CYP2E1, TNF‐α, Nf‐ĸB, IĸBα, MCP‐1, TLR4, IL‐6, IL‐1β, IL‐4, IL‐10, Nrf2, and HO‐1. β‐Actin served as a housekeeping gene. Primer sequences were designed in silico using Primer‐BLAST and validated from published studies [30] Table 2). Quantitative PCR was conducted utilizing the SYBR Green master mix on an Agilent MxPro3000 system.

**Table 2:** Gene sequences for *Rattus norvegicus* primers.

Sr. number	Gene	Forward primer (5′–3′)	Reverse primer (5′–3′)
1.	β‐Actin	TCTGTGTGGATTGGCTCTA	CTGCTTGCTGATCCACATCTG
2	CYP2E1	AACTGTCCCCGGGACCTC	GCGCTCTGCACTGTGCTTT
3.	TNF‐α	CTCTTCTGCCTGCTGCACTTG	ATGGGCTACAGCTTGTCACTC
4.	Nf‐ĸB	GCGATGTGTCCGACTCCAAA	TCTGTACTTCCTCCTTGTCTTCC
5.	IĸBα	GTACCCGGATACAGCAGCAG	CAGTCATCGTAGGGCAACTCA
6.	MCP‐1	GGTCTCTGTCACGCTTCTG	TCTTGCCAGTGAATGAGTAGC
7.	TLR4	TGGCAGTTTCTGAGTAGCCG	TCCCACTCGAGGTAGGTGTT
8.	IL‐1β	CCCTGCAGCTGGAGAGTGTGG	TGTGCTCTGCTTGAGAGGTGCT
9.	IL‐6	CATCCTCGACGGCATCTCAG	GCAGAAGAGAGCCAACCAAC
10.	IL‐4	GGTCTCAGCCCCCACCTTGC	CCGTGGTGTTCCTTGTTGCCGT
11.	IL‐10	ACTGCTAACCGACTCCTTA	TAAGGAGTCGGTTAGCAGT
12.	Nrf2	GAGAGCCCAGTCTTCATTGC	TGCTCAATGTCCTGTTGCAT
13.	HO‐1	AGAGTTTCCGCCTCCAACCA	CGGGACTGGGCTAGTTCAGG

### 2.5. Molecular Docking

The two protein targets, TNF‐α (PBD ID: 2AZ5) [31] and MCP‐1 (PBD ID: 1DOL), [32] were selected for docking analysis. The three‐dimensional structures of both proteins were retrieved from the RCSB Protein Data Bank, and preparation was performed using the Protein Preparation Wizard within the Maestro interface of the Schrödinger Suite (Schrödinger Release 2024‐2). PROPKA at pH 7.4 was used to optimize the hydrogen‐bonding network, and crystallographic water molecules located more than 5 Å from heteroatoms were removed [33]. The restrained minimization of the entire protein was performed using the OPLS4 force field, with an RMSD cutoff set at 0.3 Å [34]. The ligands NaA and NaB were converted from two‐dimensional to three‐dimensional structures by utilizing LigPrep. At physiological pH, ionization and tautomeric states were generated while maintaining the chirality of the ligands. Geometry optimization was carried out using the OPLS4 force field, and the lowest‐energy conformations were selected for docking.

The receptor grid was generated using Glide’s Receptor Grid Generation module. The total volume of 10 × 10 × 10 Å^3^ was covered on the binding site of TNF‐α and MCP‐1 in order to allow the complete conformational sampling of the ligand. For nonpolar atoms, the van der Waals scaling factor of 1.0 and a partial charge cutoff of 0.25 were assigned. The docking simulation was performed with no positional and hydrogen bond restraints to ensure unbiased binding conformations. The unbiased confirmation in the docking simulation was ensured by no positional and hydrogen bond restraints. The molecular docking was performed using Glide in Extra Precision (XP) mode by incorporating the details such as hydrophobic enclosure, desolvation, ligand strain, and hydrogen‐bonding effects [35]. After generating several poses, the topmost Glide‐scoring pose was selected by analysis. The analysis of the docking complex was performed by the Ligand Interaction Diagram tool in Maestro. The hydrogen bonds and their lengths were measured to determine the structural validity of the complex.

### 2.6. Statistical Analysis

All the in vitro experiments were performed in triplicate (*n* = 3), and in vivo experiments included six replicates in each group (*n* = 6). A one‐way ANOVA test was performed using GraphPad Prism 10.2.4. where variations were evaluated. The results are presented as the mean ± SD. The statistical significance was defined as *p* < 0.05. The statistical analysis was performed using one‐way ANOVA with Dunnett’s and Tukey’s post hoc multiple comparison test, including confidence intervals.

## 3. Results

### 3.1. Effect of NaA, NaB, and Ethanol on Cell Viability of BRL3A Cells

**Figure 1:**
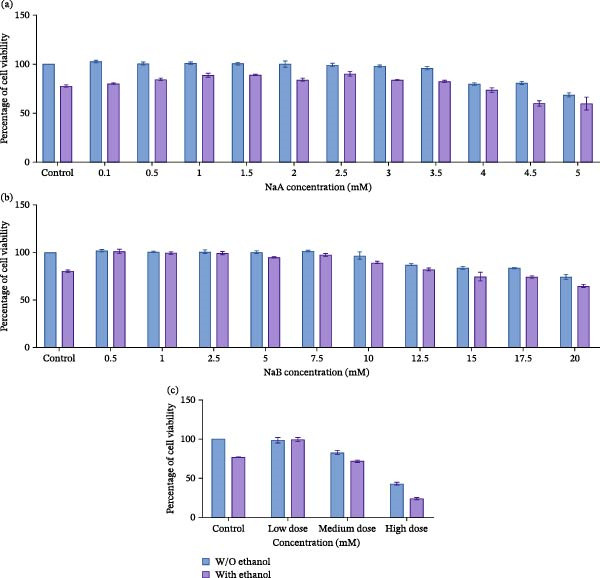
Dose‐dependent MTT assay (cytotoxicity assay) of (a) NaA, (b) NaB, and (c) their combination performed on BRL3A cells. A gradual decline in cell viability was observed in cells without ethanol treatment. The cells exposed to ethanol exhibited more pronounced cell death, whereas cotreatment of ethanol with NaA, NaB, and their combination reduced the ethanol‐induced cytotoxicity. Values are represented as mean ± SD (*n* = 3). (a) Significance was calculated using one‐way ANOVA with Dunnett’s multiple comparisons, where the significance of NaA concentration of 0–1.5 mM was *p* > 0.05, whereas 0 and 2–3.5 mM was *p* < 0.01 and 0 and 4–5 mM was *p* < 0.0001. (b) The significance of NaB concentration of 0–7.5 mM was *p* > 0.05, whereas 0 and 10 mM was *p* < 0.01 and 0 and 12.5–20 mM was *p* < 0.0001. (c) The significance of combination group of NaA and NaB for concentration of 0 and 0.1 + 1 mM was *p* > 0.05, whereas 0 and 1 + 2.5 mM was *p* < 0.01 and 0 and 2.5 + 7.5 mM was *p* < 0.0001.

The MTT assay was employed to evaluate the cytotoxicity of NaA, NaB, and their combination alone and against ethanol (250 mM). The results demonstrated the concentration‐dependent decline in cell viability (Figure 1). The treatment with 3 mM NaA and 10 mM NaB alone maintained almost 100% viability. However, against alcohol treatment, the effective doses of NaA and NaB were 1.5 mM and 5 mM, respectively, and therefore, these doses were selected as a treatment dose. Further, NaA and NaB exhibited moderate toxicity at concentrations above 5 mM and 20 mM, respectively, resulting in viability of ~59% and 64% in the ethanol cotreatment groups. For the combination of NaA and NaB, three doses were tested: the low dose (0.1 + 1 mM), medium dose (1 + 2.5 mM), and high dose (2.5 + 7.5 mM) (Figure 1). Among these, the low dose showed the highest viability in both the presence and absence of ethanol cotreatment groups.

### 3.2. NaA, NaB, and Their Combination Attenuate Oxidative Stress and Nuclear Damage

**Figure 2:**
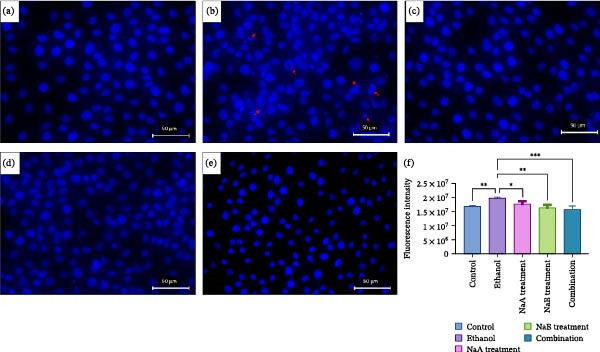
Nuclear morphology was visualized by DAPI staining of BRL3A cells (at 40x magnification) in the presence and absence of ethanol. The groups included are (a) control, (b) ethanol (250 mM), (c) NaA (1.5 mM), and 250 mM of ethanol, (d) NaB (5 mM) and 250 mM of ethanol, and (e) combination (NaA) 0.1 mM + (NaB) 1 mM) and 250 mM of ethanol. (f) Quantification of DAPI fluorescence intensity. Values are represented as mean ± SD, and significance was calculated using one‐way ANOVA with Dunnett’s multiple comparisons where  ^∗^ *p* < 0.05,  ^∗∗^ *p* < 0.01, and  ^∗∗∗^ *p* < 0.001. (*n* = 3) (scale bar = 50 μm).

**Figure 3:**
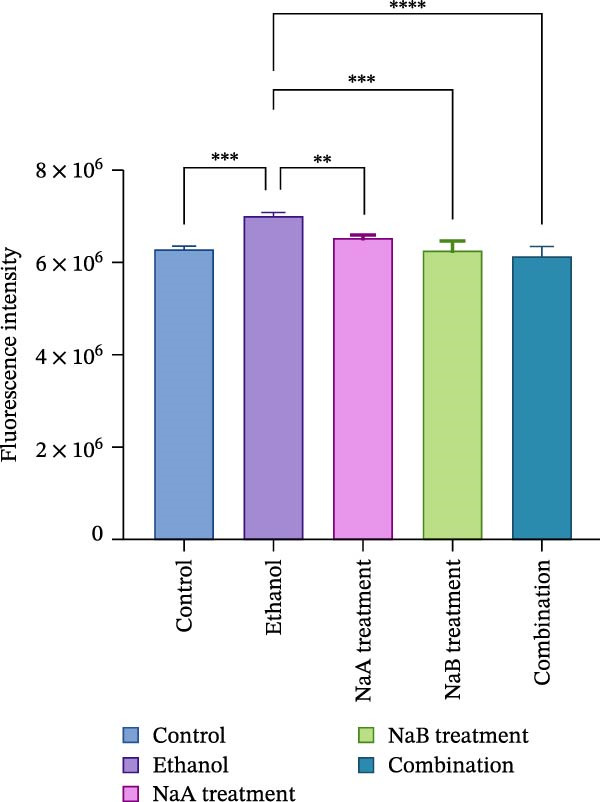
Quantification of ROS by DCFDA fluorescence intensity in BRL3A cells in presence of ethanol (250 mM) in all experimental groups and cotreatment of NaA (1.5 mM), NaB (5 mM), combination (NaA) 0.1 + (NaB) (1 mM). Values are represented as mean ± SD and significance was calculated using one‐way ANOVA with Dunnett’s multiple comparisons where,  ^∗∗^ *p* < 0.01,  ^∗∗∗^ *p* < 0.001 and  ^∗∗∗∗^ *p* < 0.0001. Post hoc comparisons using Tukey test indicated significant differences between groups, with 95% confidence intervals documented (*n* = 3).

The incorporation of ethanol inflicts oxidative stress and affects nuclear morphology, as observed and measured by DCFDA and DAPI staining techniques. The cells exposed to only ethanol exhibited an irregular structure and nuclear fragmentation (Figure 2b), which was validated by increased fluorescence intensity (Figure 2f). Conversely, the control group had more stable (Figure 2a) and preserved nuclei, and identical observations were made for treatment groups (Figure 2c–e). Furthermore, the cotreatment groups of ethanol and NaA, NaB, and their combination, respectively, showed a marked reduction in DCFDA fluorescence intensity relative to the ethanol‐only group (Figure 3). Further, a total of 10 distinct fields with over 500 nuclei were evaluated to confirm the results.

### 3.3. NaA, NaB, and Their Combination Reduced Cellular Damage by Maintaining Cell Membrane Integrity

**Figure 4:**
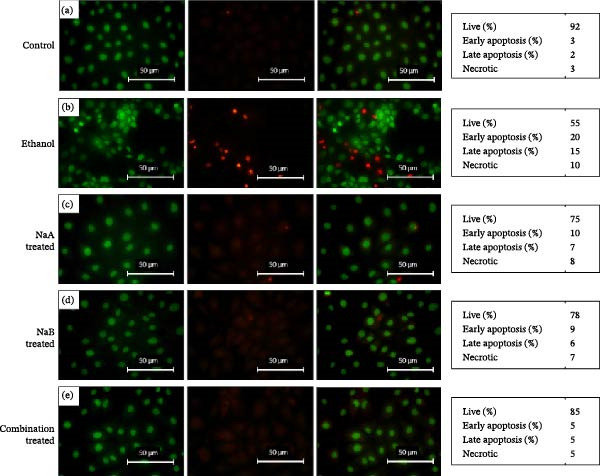
Cellular damage was assessed by AO/EtBr staining of BRL3A cells (at 40x magnification). (a) Represents positive control for analyzing the experimental groups. Further, exposure to 250 mM ethanol (b) was used, and treatments included ethanol (250 mM) and NaA (1.5 mM), ethanol (250 mM) and NaB (5 mM) a combination (NaA) 0.1 mM + (NaB) 1 mM (c–e) with ethanol (250 mM). The quantification was performed by analyzing 10 random field and the percentage of live, early apoptosis, late apoptosis, and necrosis were calculated using ImageJ software. Green and red fluorescence signals were thresholded to identify viable and nonviable cells, whereas the apoptotic stages and necrosis were distinguished based on nuclear morphology. The live cells were identified by uniform green nuclei. The indication of bright green with condensed chromatin revealed early apoptotic stage. Further, late apoptosis was identified by orange/red fluorescence with fragmented nuclei. Finally, necrotic nuclei exhibited uniformly red nuclei. (*n* = 3) (sale bar 50 µM).

Dual staining with AO/EtBr is employed to visualize cellular damage. Damaged cells absorb EtBr and emit red fluorescence owing to the compromised membrane integrity. Cells exposed to ethanol demonstrated substantial damage (Figure 4). Compared to the ethanol‐only group, the administration of NaA, NaB, and their combination attenuated the ethanol‐induced damage (Figure 4).

### 3.4. NaA, NaB, and Their Combination Mitigate Hepatic Inflammation and Oxidative Stress‐Related Gene Expression In Vitro

**Figure 5:**
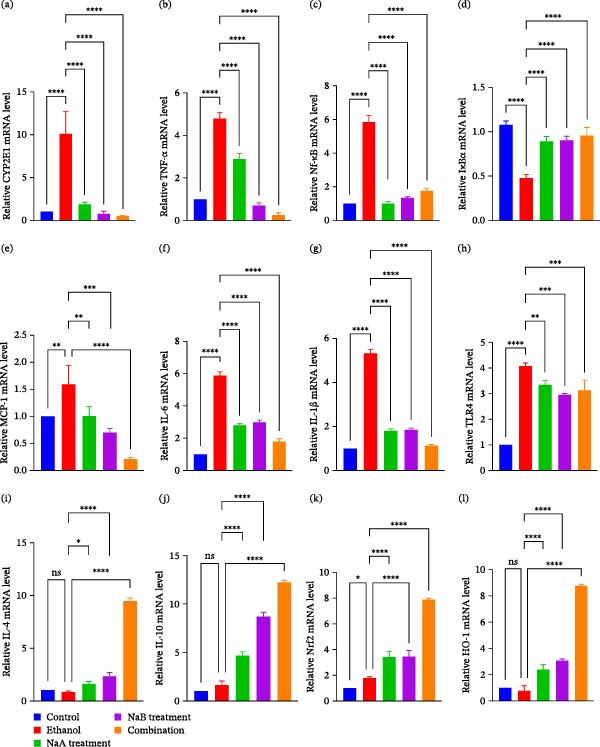
NaA, NaB, and NaA + NaB help reduce hepatic inflammation and has an antioxidant activity in BRL3A cells against ethanol (250 mM). Gene expression over β‐actin in vitro in different treatment groups showing (a) CYP2E1, (b) TNF‐α, (c) Nf‐ĸB, (d) IκBα, (e) MCP‐1, (f) TLR4, (g) IL‐6, (h) IL‐1β, (i) IL‐4, (j) IL‐10, (k) Nrf2, and (l) HO‐1. Values are represented as mean ± SD and significance was calculated using one‐way ANOVA with Dunnett’s multiple comparisons where ^ns^ *p* > 0.05,  ^∗^ *p* < 0.05,  ^∗∗^ *p* < 0.01,  ^∗∗∗^ *p* < 0.001 and  ^∗∗∗∗^ *p* < 0.0001. Post hoc comparisons using Tukey test indicated significant differences between groups, with 95% confidence intervals documented (*n* = 3).

Gene expression studies on BRL3A cells confirmed that NaA, NaB, and their combination confer protection against alcohol‐induced hepatic damage. Cells exposed to ethanol exhibited a significant elevation in the CYP2E1 expression (10.13 ± 2.57) (Figure 5a). Nevertheless, cotreatment with ethanol and respective treatments of NaA, NaB, or their combination notably reduced CYP2E1 levels, with the combination group showing a significant decrease to 0.50 ± 0.04. Ethanol‐treated cells also demonstrated increased levels of pro‐inflammatory cytokines TNF‐α, IL‐1β, IL‐6, and MCP‐1, along with diminished expression of the IκBα subunit, thereby activating the TLR4‐mediated NF‐κB signaling pathway. Among the treatments, groups of ethanol with the combination of NaA and NaB most effectively inhibited NF‐κB signaling, reducing pro‐inflammatory cytokine expression more than either NaA or NaB alone (Figure 5b–h). Additionally, NaA, NaB, and their combination exhibited anti‐inflammatory and antioxidant effects by elevating anti‐inflammatory cytokines IL‐10, IL‐4, Nrf2, and HO‐1, thereby restoring redox homeostasis (Figure 5i–l).

### 3.5. NaA, NaB, and Their Combination Reduce Alcohol‐Induced Liver Damage In Vivo

#### 3.5.1. NaA, NaB, and Their Combination Regulate the Body Weight, Liver Morphology, and Liver‐to‐Body Weight Ratio

**Figure 6:**
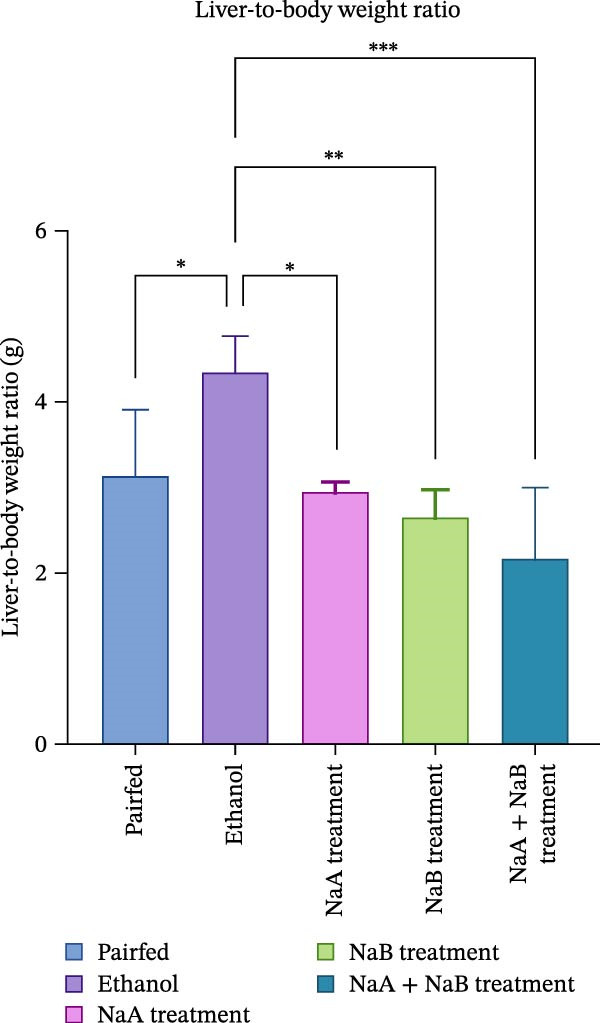
Body‐to‐weight ratio of male Wistar rats collected after study completion. Values are represented as mean ± SD and significance was calculated using one‐way ANOVA with Dunnett’s multiple comparisons where,  ^∗^ *p* < 0.05,  ^∗∗^ *p* < 0.01,  ^∗∗∗^ *p* < 0.001. Post hoc comparisons using Tukey test indicated significant differences between groups, with 95% confidence intervals documented (*n* = 6).

**Figure 7:**
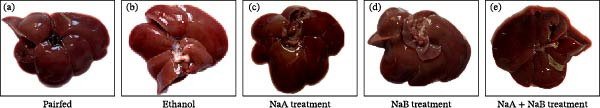
Representative images of rat liver. (a) Pair‐fed group displaying normal size and lobular structure. (b) Ethanol group exhibiting discoloration and friable tissue appearance. (c) NaA treatment group partially restoring normal liver appearance. (d) NaB treatment group showing improved morphological structure. (e) Combination treatment group demonstrating complete restoration of morphology, resembling the control group.

Both the PF group, which is used as a control group (isocaloric diet group), and the treatment groups gained body weight gradually over the 5 weeks (Table 3). The ethanol‐treated group (E) exhibited a notable increase in weight gain, suggesting disrupted metabolism. After 5 weeks, the liver‐to‐body weight ratio in the ethanol‐fed group was significantly higher than in the treatment groups (Figure 6). No significant differences were observed for the PF group. The morphology of the liver was significantly compromised in the ethanol‐treated group; however, it was restored in the treatment groups of NaA, NaB, and their combination with ethanol cotreatment, particularly within the combination group (Figure 7).

**Table 3:** Body weight of rodents (g) during 5 weeks of pair‐fed, ethanol‐fed, NaA, NaB, and NaA + NaB treatment groups (mean ± SD), (*n* = 6).

Body weight (g)	Week 1	Week 2	Week 3	Week 4	Week 5
Pair fed	177 ± 7	196 ± 6	245 ± 1	266 ± 5.50	278 ± 17.61
Ethanol fed	187 ± 11.23	210 ± 13	250 ± 15.04	270 ± 11.50	290 ± 12.56
NaA treated	166 ± 1.73	180 ± 15.53	195 ± 11.53	205 ± 9.71	225 ± 9.45
NaB treated	188 ± 7.02	205 ± 6.24	210 ± 9.07	220 ± 13.42	253 ± 12.12
Combination treated	186 ± 2.51	221 ± 1.52	219 ± 3.21	225 ± 7.02	254 ± 3.05

#### 3.5.2. NaA, NaB, and Their Combination Lowered the Liver Injury Markers

The biochemical evaluation revealed altered liver injury markers in ethanol‐treated rats compared to those in their respective PF groups. The diseased group showed increased triglycerides, total cholesterol, AST, and GGT levels, indicating fat accumulation and inflammation in the liver (Table 4).

**Table 4:** The serum biochemical parameters of pair‐fed, ethanol, and treatment groups.

Blood parameters	Pair fed	Ethanol fed	NaA treated	NaB treated	Combination treated
Cholesterol	70.45 ± 0.79^a^	90.55 ± 1.04^b^	81.60 ± 3.05^c^	82.15 ± 1.36^c^	79.10 ± 2.05^ac^
Triglycerides	70.95 ± 0.70^a^	92.97 ± 0.40^b^	79.63 ± 0.85^c^	80.87 ± 0.64^c^	73.68 ± 0.48^a^
ALT	31.59 ± 0.10^a^	45.97 ± 0.68^b^	35.47 ± 0.57^c^	30.66 ± 0.71^a^	27.45 ± 1.32^a^
AST	60.34 ± 1.30^a^	91.86 ± 1.61^b^	58.79 ± 0.57^a^	55.17 ± 1.02^a^	50.75 ± 0.56^a^
GGT	50.52 ± 0.59^a^	65.80 ± 0.39^b^	55.15 ± 0.27^c^	52.04 ± 0.14^ac^	50.59 ± 0.43^a^

*Note:* The values are displayed in (mean ± SD) (*n* = 6). The superscript letters (a–c) indicate significant differences (*p* < 0.05) among groups as determined by one‐way ANOVA with Tukey’s post hoc test.

#### 3.5.3. NaA, NaB, and the Combined Group Showed a Protective Effect and Maintained Liver Architecture

**Figure 8:**
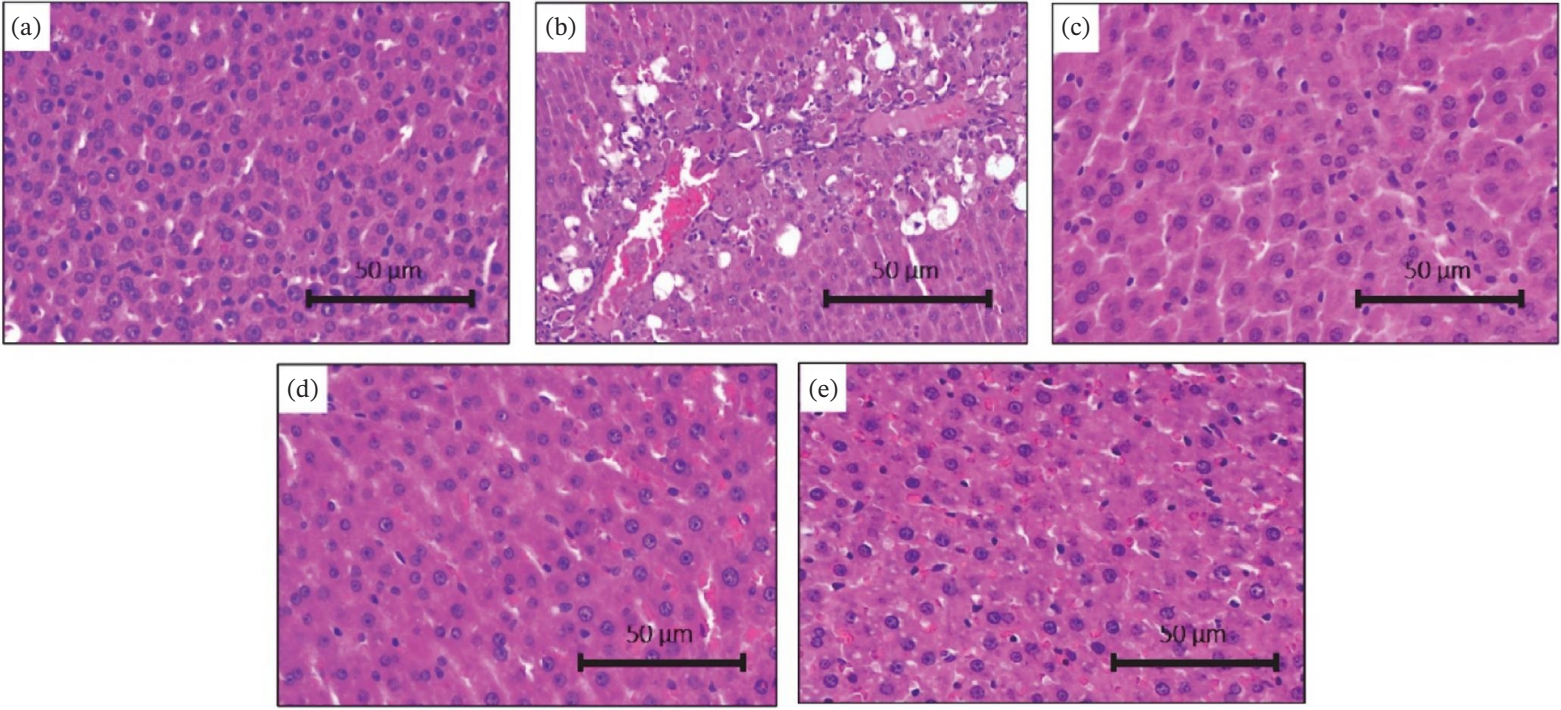
Histological evaluation of liver by hematoxylin and eosin staining. (a) Pair‐fed group with normal morphology. (b) Ethanol‐fed rats displaying ballooning degeneration. (c) NaA treatment group showed improved morphology of hepatocytes. (d) NaB treatment rodents also displayed improved cell integrity. (e) Pronounced improvement was observed in hepatocytes in combination treatment group. Images are taken at 40x magnification, and the scale bar depicts 50 μm.

**Figure 9:**
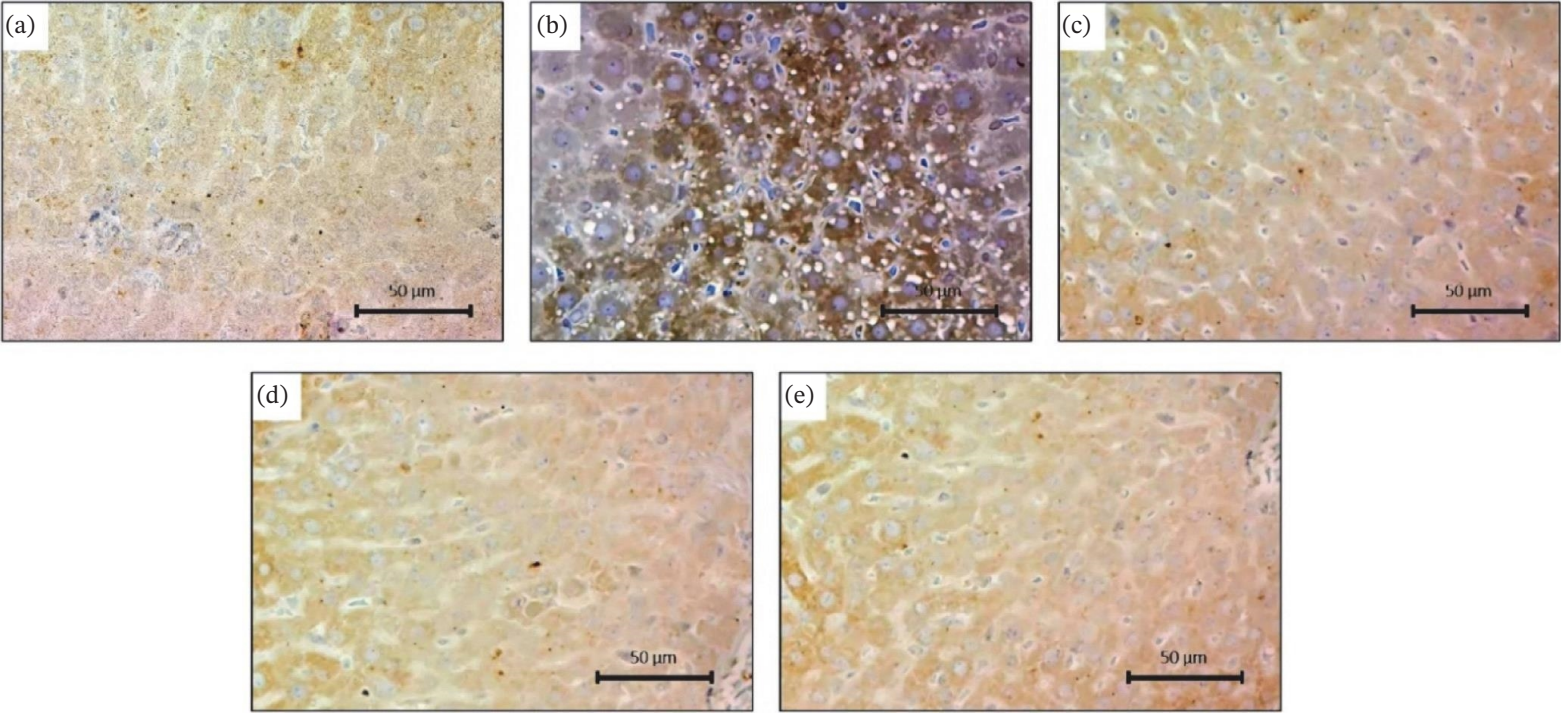
Immunohistochemistry staining of CYP2E1 in liver sections. The rat liver section at 40x magnification stained using anti‐CYP2E1 antibody showing (a) pair‐fed with standard features, (b) increased positive staining in ethanol‐fed rats, and decreased in (c) NaA, (d) NaB, and (e) combination groups. (*n* = 6) (scale bar 50 µM).

Clear signs of hepatic steatosis were observed in ethanol‐fed Wistar rats. Additionally, the cytoplasmic macrovesicular structure (Figure 8b) was seen in the ethanol group. The treatment groups had intact hepatocytes, indicating reduced inflammation and fat accumulation, which was confirmed in H&E staining (Figure 8c–e). The liver cell morphology and immunohistostaining of CYP2E1 were normal in the case of the PF group (Figures 8a and 9a). Likewise, the liver section stained with immunohistochemical markers for CYP2E1 revealed a prominent CYP2E1‐positive area primarily in the centrilobular regions of the ethanol‐fed group (Figure 9b). The treatment groups of NaA, NaB, and their combination groups with ethanol cotreatment showed a decrease in CYP2E1 immunostaining intensity in the pericentral area (Figure 9c–e). Notably, the combination group had a dual effect on restoring the overall hepatocyte morphology.

#### 3.5.4. NaA, NaB and Their Combination Suppress Hepatic Inflammation and Upregulate Antioxidant Genes In Vivo

**Figure 10:**
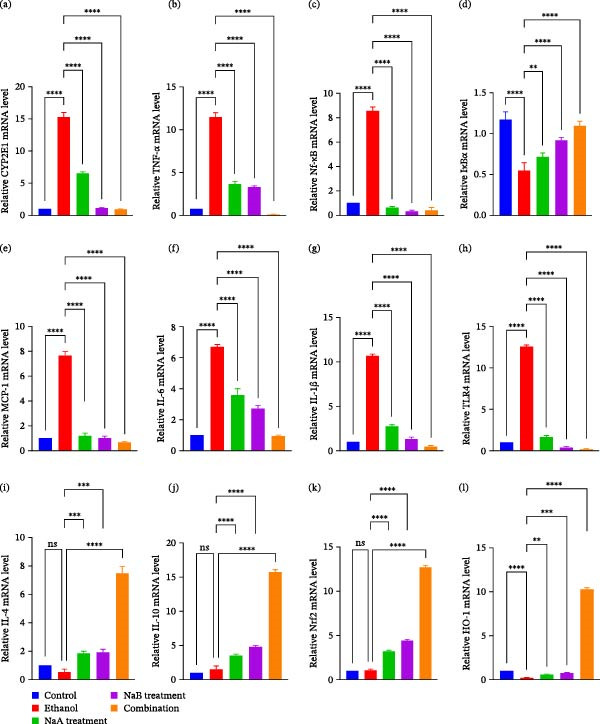
NaA, NaB, and combination group demonstrated a hepatoprotective effect in rats against alcohol‐induced damage. Gene expression over β‐actin in vivo in different treatment groups showing (a) CYP2E1, (b) TNF‐α, (c) Nf‐ĸB, (d) IĸBα, (e) MCP‐1, (f) TLR4, (g) IL‐6, (h) IL‐1β, (i) IL‐4, (j) IL‐10, (k) Nrf2, and (l) HO‐1. Values are represented as mean ± SD and significance was calculated using one‐way ANOVA with Dunnett’s multiple comparisons, where ^ns^ *p* > 0.05,  ^∗∗^ *p* < 0.01,  ^∗∗∗^ *p* < 0.001 and  ^∗∗∗∗^ *p* < 0.0001. Post hoc comparisons using Tukey test indicated significant differences between groups, with 95% confidence intervals documented (*n* = 6).

Research on gene expression in liver tissue samples from living subjects revealed notable variations in cytokine levels. In particular, CYP2E1 levels were tenfold higher than those observed in the corresponding treatment groups (Figure 10a). A key chemokine, MCP‐1, played a significant role in attracting pro‐inflammatory cytokines. Gene expression analysis indicated that MCP‐1 was significantly upregulated (8.00 ± 0.08) and contributed substantially to inflammation in Wistar rats fed with ethanol (Figure 10e). The primary pro‐inflammatory cytokine, TNF‐α, was also upregulated (11.44 ± 0.53) in ethanol‐fed rats compared to the PF group (Figure 10b). In contrast, the treatment groups of NaA, NaB, and their combination group where ethanol cotreatment was given exhibited decreased levels of MCP‐1 and TNF‐α (Figure 10b,e). Further, other pro‐inflammatory cytokines such as IL‐1β and IL‐6 were reduced in SCFA‐treated groups compared to diseased groups (Figure 10f–h). The IκBα subunit was upregulated in ethanol and NaA, NaB, and the combined group, respectively, reflecting the downregulation of the NF‐κB signaling pathway (Figure 10c,d). Overall, the profound effect of the combination of the NaA and NaB group was observed in statistics of gene expression, indicating the synergistic action against alcoholic hepatotoxicity.

Alcohol consumption in rats leads to oxidative stress in the cells, resulting in excessive production of ROS. NaA, NaB, and their combination modulated the activity of the transcription factor Nrf2, predominantly in the combination treatment group (3.00 ± 0.17), which binds to antioxidant response elements (AREs) and activates HO‐1. This activation facilitates the release of antioxidant and anti‐inflammatory cytokines. The NaA, NaB, and their combination stimulate a defensive response by increasing IL‐4, IL‐10, Nrf2, and HO‐1 (Figure 10i–l), thereby counteracting the damage caused by ethanol.

#### 3.5.5. NaA, NaB, and Their Combination Show Hepatoprotective Effect Against Alcohol‐Induced Damage Measured by ELISA in Rats

**Figure 11:**
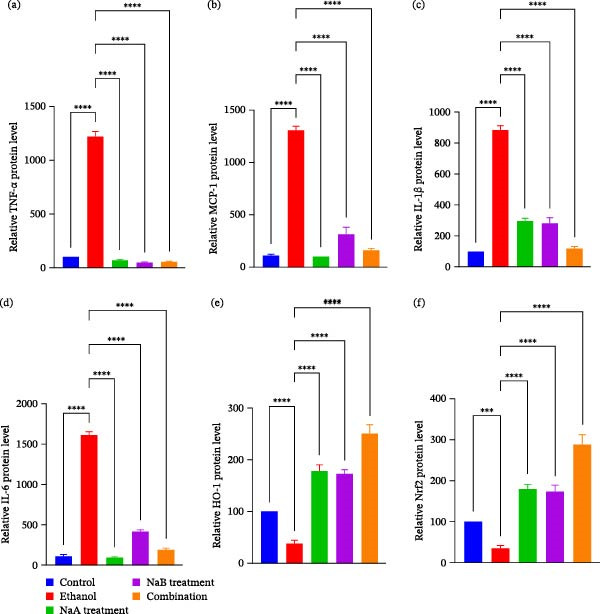
Protein expression profiles of (a) TNF‐α, (b) MCP‐1, (c) IL‐1β, (d) IL‐6, (e) HO‐1, and (f) Nrf2 in rat brain tissue samples. Values were represented as mean ± SD, and significance was calculated using one‐way ANOVA with Dunnett’s multiple comparisons, where  ^∗∗∗^ *p* < 0.001 and  ^∗∗∗∗^ *p* < 0.0001. Post hoc comparisons using Tukey test indicated significant differences between groups, with 95% confidence intervals documented (*n* = 6).

The protein expression in Wistar rats was analyzed utilizing an ELISA. TNF‐α levels were considerably elevated in the diseased cohort (ethanol‐fed group) in comparison to those in the control group. A comparable trend was observed with other pro‐inflammatory cytokines, including IL‐1β, IL‐6, and the chemokine MCP‐1. Nonetheless, a significant reduction in these proteins was noted within the treatment groups, particularly in the combination group receiving NaA and NaB with ethanol. A similar pattern of improved protein expression was evident in the gene expression of Nrf2 and HO‐1, thereby corroborating the overall findings (Figure 11).

### 3.6. Molecular Docking

**Figure 12:**
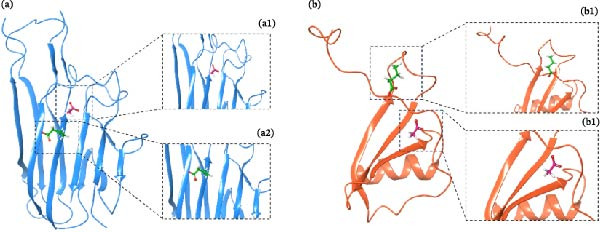
The interactions of NaA and NaB with macromolecules. (a) Stable interaction between TNF‐α protein and (a1) NaA (pink) and (a2) NaB (green). (b) Stable interaction between MCP‐1 protein with (b1) NaB (green) and (b2) NaA (pink).

**Figure 13:**
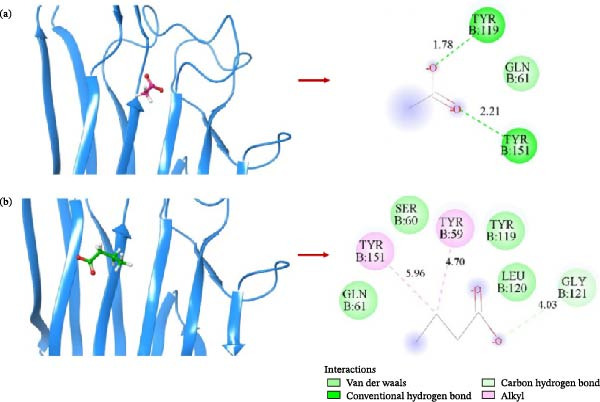
The TNF‐α protein interacts with (a) NaA (pink) and (b) NaB (green) through multiple stabilizing contacts. The stability of each ligand–protein complex is primarily supported by hydrogen bonds and weak intramolecular forces, including van der Waals interactions.

**Figure 14:**
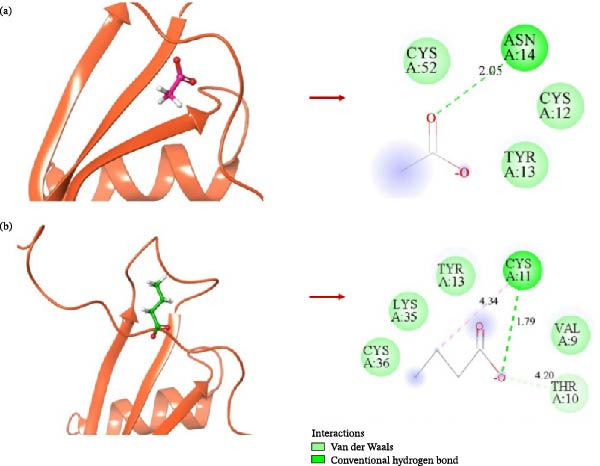
The MCP‐1 protein interacts with (a) NaA (pink) and (b) NaB (green) through multiple stabilizing contacts. The stability of each ligand–protein complex is primarily supported by hydrogen bonds and weak intramolecular forces, including van der Waals interactions.

The molecular docking analysis of NaA and NaB ligands against TNFα and MCP‐1 revealed that the ligands interacted with the active sites of both proteins and the surface interactions of NaA and NaB with their respective target proteins indicate that the ligands bind to the active sites and collectively form a strong interacting interface (Figure 12) through the formation of hydrogen bonds, Pi‐alkyl interactions, and van der Waals interactions (Figures 13a,b and 14a,b). Due to the limited molecular structures of NaA and NaB (C_2_H_3_NaO_2_ and C_4_H_7_NaO_2_), only a limited number of interactions were observed with the targeted proteins. The binding energy of the NaA–TNF‐α complex was −2.547 kcal/mol and that of the NaB–TNF‐α complex was −3.21 kcal/mol. NaA established stable hydrogen bonds with TYR119 and TYR151, with bond lengths of 1.78 Å and 2.21 Å, respectively. Van der Waals interactions were also observed at the GLN61 residue in the NaA–TNF‐α complex. Similarly, NaB, a ligand with a C_4_ carbon structure, exhibited pi‐alkyl interactions with TYR151 and TYR59 residues, along with van der Waals interactions at GLY121, LEU120, TYR119, SER60, and GLN61 of the TNF‐α macromolecule. Furthermore, the NaA–MCP‐1 and NaB–MCP‐1 complexes exhibited binding energies of −1.871 kcal/mol and −1.861 kcal/mol, respectively. The stability of the NaA–MCP‐1 complex was attributed to hydrogen bond formation at ASN14 (2.05 Å), while a stable hydrogen bond at CYS11 (1.79 Å) supported the stability of the NaB–MCP‐1 complex. Additional interactions included C─H interaction at THR10 and alkyl interactions at CYS11. The glide energies for the NaA–TNF‐α and NaB–TNF‐α complexes were −11.324 kcal/mol and −12.424 kcal/mol, respectively. For the NaA–MCP‐1 and NaB–MCP‐1 complexes, the glide energies were −9.744 kcal/mol and −10.104 kcal/mol, respectively.

## 4. Discussion

Prolonged alcohol consumption is regarded as a major cause of health complications, affecting vital organs. As the liver is the primary site of ethanol metabolism, it is subjected to substantial damage from ethanol, eventually leading to progressive stages of alcoholic steatosis, hepatitis, fibrosis, and cirrhosis [36, 37]. Extensive alcohol intake elicits a range of cellular responses, including oxidative stress, inflammation, and nutritional imbalances [38]. The SCFA intervention, particularly of NaA and NaB, can interact with inflammatory pathways and mitigate oxidative stress across various diseases, including ALD [39, 40]. Recent studies have emphasized their role in liver diseases, neuroinflammation, and metabolic dysfunction. The evidence obtained from in vitro and in vivo studies substantiates their anti‐inflammatory and antioxidant properties, further affirming their prospective therapeutic application.

NaA and NaB exert proliferative capacity while protecting cells against alcohol‐induced stress, leading to restoration of normal cellular function. The cytotoxicity evaluated of NaA and NaB with their combined group exhibited a dose‐dependent effect on the BRL3A cell line. A study demonstrated that up to a 20 mM concentration of NaB was non‐toxic and did not compromise HepG2 cell viability [41]. Likewise, NaA did not affect viability up to a 4 mM concentration of BRL3A cells, which were analyzed for lipid metabolism via the AMPK signaling pathway [42]. Our results followed a similar pattern, with NaA, NaB, and their combination enhancing cell viability. Ethanol treatment reduced the cell viability and significantly induced oxidative damage in the cells, which was partially restored in the NaA‐ and NaB‐treated group, while the group receiving the combined treatment markedly decreased ROS‐induced cellular and nuclear damage.

The alcohol‐related hepatic damage develops through the dynamic interaction of oxidative stress and inflammatory cytokines. Pathogen‐derived stimuli, stress signals, and inflammatory cytokines could achieve the activation of NF‐κB [43]. The expression of NF‐κB is further implicated in the generation of essential pro‐inflammatory cytokines, establishing a closely interconnected feedback mechanism of NF‐κB activation. It is imperative to disrupt this feedback loop to mitigate inflammation and prevent disease progression. Recent research has indicated that NaA and NaB directly modulate hepatic immune cells, diminish the release of pro‐inflammatory factors such as IL‐1β, TNF‐α, and IL‐1 within the liver, and enhance the expression of anti‐inflammatory factors IL‐4 and IL‐10 [13]. The present study corroborates these findings, demonstrating that NaA, NaB, and their combination may significantly reduce cytokine production, with a key component of the NF‐κB pathway attainably inhibited by SCFA.

Further, the SCFAs could empirically inhibit NF‐κB reduce neutrophil infiltration and oxidative stress. Among all treatment groups, NaB and the combination of both SCFAs most effectively inhibited NF‐κB compared to the acetate treatment group, confirmed by H&E staining and immunohistochemical staining. Neutrophil infiltration is a key hallmark of inflammation in hepatocytes, and it was less evident in the treatment groups compared to the ethanol‐treated group. Similarly, one of the vital pathways, the CYP2E1‐dependent microsomal ethanol oxidizing system, is an essential pathway of ethanol metabolism in the liver, which is induced by persistent ethanol consumption. The previous studies support that ethanol‐induced overexpression of CYP2E1 is a critical mediator, possibly through oxidative stress and ethanol‐induced inflammatory response [13]. In the present study, the ethanol‐fed group exhibited an extensive expression of CYP2E1, as confirmed by the intensity of staining. Conversely, this expression was observed to be less prominent in the treatment groups.

Elevation in hepatic biomarkers can assist in classifying liver damage. GGT is one of the main serum biomarkers linked to alcohol‐related liver disease. Likewise, intensified AST and AST levels indicate necrosis and inflammation in hepatocytes. Furthermore, alcohol intake can impair lipolysis and result in lipid accumulation that is reflected as increased levels of TG and cholesterol in the serum [44]. NaA and NaB are versatile in nature, providing them an advantage to intervene in many physiological processes. Our findings indicate that the NaA and NaB combined group markedly reduced the liver injury serum biomarkers and improved the lipid profile, GGT, AST, and ALT levels.

The experimental outcomes of NaA and NaB against alcohol‐induced cellular impairment showed promising results, a further validation was provided by computational methodologies. Molecular docking substantiated the hypothesis regarding the protective effect of NaA and NaB by forming a stable complex with TNF‐α and MCP‐1. NaA and NaB are small molecules, consisting of two and four carbon units, making them constrained to form various types of bonds with macromolecules. As documented by [30] TNF‐α and [45] MCP‐1, they form stable complexes due to strong hydrogen bond affinity. Likewise, in the current study, NaA and NaB interacted with identical residues and established a stable hydrogen bond. Another computational method, molecular dynamics (MD) simulation, provides the likely potential of ligands to sustain interaction with macromolecules. However, due to the limited size of NaA and NaB, MD simulations were not supported by the software.

The treatment of NaA and NaB could synergistically demonstrate a superior hepatoprotective effect in alcohol‐induced liver damage compared to individual treatments. The efficacy of SCFAs is achieved by targeting major pro‐inflammatory cytokines, downregulating CYP2E1, and curtailing cellular and nuclear oxidative distress. These findings support the development of this combination as a new epoch therapeutic strategy for alcohol‐induced hepatotoxicity.

## 5. Conclusion

Ethanol misuse poses significant risks to liver function by inflicting hepatocellular damage and disrupting physiological processes. Excessive alcohol exposure may result in enhanced activity of NF‐κB and CYP2E1 upregulation, ultimately increasing oxidative stress. The possibly activated NF‐κB signaling cascade releases pro‐inflammatory cytokines, which in turn accelerate inflammatory responses. The study is based on controlled experimental models, which may not entirely encapsulate the complexity of human alcoholic liver disease. Furthermore, the sample size and experimental duration might restrict the generalizability and long‐term interpretive capacity of the findings. Differences in SCFA metabolism, gut–liver axis interactions, and systemic physiology in humans necessitate careful consideration. In this investigation, cotreatment with NaA and NaB conserved the normal morphology of hepatocytes and aggravated the antioxidative and anti‐inflammatory cytokines. These findings suggest SCFAs as promising therapeutic candidates for attenuating hepatotoxicity and unfold the route for exploring translational and clinical research. To enhance mechanistic understanding, future investigations ought to include direct validation of NF‐κB signaling pathway activity utilizing targeted molecular assays such as nuclear translocation analysis or DNA‐binding assays.

## Author Contributions

Manali Patel designed the study, conducted the cell line experiments, and performed all analyses. Hiral Aghara, Prashsti Chadha, and Harshrajsinh Solanki carried out the animal study. Manali Patel and Palash Mandal contributed to the drafting of the initial manuscript. Manali Patel, Hiral Aghara, Prashsti Chadha, Harshrajsinh Solanki, and Palash Mandal reviewed and edited the manuscript.

## Funding

No funding was received for conducting the experimental work.

## Conflicts of Interest

The authors declare no conflicts of interest.

## Data Availability

The data that support the findings of this study are available from the corresponding author upon reasonable request.
